# *Pneumocystis* Pneumonia: Still a Serious Disease in Children

**DOI:** 10.34763/devperiodmed.20192303.159162

**Published:** 2019-10-27

**Authors:** Magdalena Zakrzewska, Renata Roszkowska, Mateusz Zakrzewski, Elżbieta Maciorkowska

**Affiliations:** 1 Department of Developmental Age Medicine and Pediatric Nursing, Medical University of Białystok, Białystok, Poland; 2 Department of Pediatrics and Pulmonary Diseases, The Children’s University Hospital, Białystok, Poland

**Keywords:** children, *Pneumocystis* pneumonia, symptoms, treatment

## Abstract

Pneumocystis carinii pneumonia is a common opportunistic respiratory infection among children with human immunodeficiency virus and a weakened immune system. The primary infection in immunocompetent patients may be asymptomatic, whereas fever, shortness of breath, night sweats, nonproductive (dry) cough, pneumonia, progressive respiratory distress and apnea are cardinal symptoms of full-blown pneumocystis pneumonia. The diagnosis can be confirmed by histochemical staining of biological specimens or, recently, by polymerase chain reaction. International recommendations indicate that the drug of choice is the intravenously administered trimethoprim-sulfamethoxazole combination. Early diagnosis and appropriate treatment reduce the mortality of the disease. This article briefly highlights the epidemiology of Pneumocystis pneumonia, its diagnosis and therapeutic options in the pediatric population.

## Introduction

*Pneumocystis jirovecii* causes opportunistic respiratory infections in young children with weakened immune systems. Individuals with HIV infection, neoplastic disorders, or patients receiving long-term corticosteroid therapy are all commonly affected [1]. *Pneumocystis jirovecii*, formerly known as *Pneumocystis carinii*, is an eukaryotic fungal pathogen responsible for *Pneumocystis carinii* pneumonia (PCP). PCP was for the first time described in 1940 as interstitial plasma cell pneumonia [2]. Pneumocystis can be detected in bronchoalveolar lavage (BAL), tracheal aspirate, sputum, or gastric aspirate using polymerase chain reaction (PCR) or during microscopic examination. Wakefield et al. described the diagnosis of *Pneumocystis jirovecii* infection by PCR for the first time in the early 1990s [3]. Among an array of authors [4, 5, 6, 7], Gupta et al. were the first to suggest that PCR assay is likely to play a critical role in confirming the diagnosis of PCP [5]. Of note, PCR was used early for the identification of *Pneumocystis jirovecii* mutations associated with drug resistance [4].

Furthermore, the colonization of the respiratory tract by the fungus without manifestation of clinical symptoms may lead to an overlooked damage of the lungs [6]. The mortality rate of PCP without treatment in patients with weakened immune systems is nearly 100%. Interestingly, the morbidity is lower in patients with acquired immune deficiency syndrome compared to oncologic patients [7].

## Epidemiology and symptoms

The patient’s first exposure to *Pneumocystis jirovecii* usually occurs in early childhood. Immunocompetent children can be a reservoir of *Pneumocystis* DNA in

their respiratory tract and their primary infection is generally asymptotic. Interestingly, an epidemiological study published in 2000 and focused on newly acquired infections indicated that the most probable route of infection is direct person-to-person transmission [8]. In the study by Pifer et al. on a cohort of immunocompetent infants, the seroconversion rate obtained in children at 4 years of age reached 75% [9]. Both *Pneumocystis* colonization and subclinical infection seem to be associated with the actual condition of the immune system [10]. In a cautionary tale, Vargas et al. reported on a possible relationship between sudden infant death syndrome and *Pneumocystis* colonization. This group of researchers performed autopsy studies of the lungs and found that 3% of the infants were positive for *Pneumocystis* [11]. Yet, in a following study, they suggested that *Pneumocystis* colonization plays an unclear role in the syndrome [12].

The principal risk factors for PCP are human immunodeficiency virus infection, prolonged use of corticosteroids, certain cancer types, chemotherapy, transplantation procedures (especially of the heart or lung/s) , and rheumatic diseases [13, 14]. The risk of PCP occurrence has been related to defects in T-cell immunity. For instance, Calderón et al. presented higher CD4+ counts in non-immunosuppressed patients colonized with *Pneumocystis* than in immunosuppressed ones [15]. Another group reported that mice with a decrease in the CD4+ count acquire persistent *Pneumocystis* infection [16]. The long-term use of corticosteroids reduced the CD4 lymphocyte count, particularly in the lungs [17]. In the majority of non-acquired immunodeficiency syndrome (AIDS) cases, PCP is associated with the Wiskot-Aldrich syndrome and X-linked Hyper-IgM Syndrome [18].

The classical presentation of *Pneumocystis* infection includes symptoms of fever, shortness of breath, pneumonia, progressive respiratory distress, and apnea. Infants and young children may also demonstrate less common symptoms, such as decreased appetite, malaise, and cyanosis. Cases being co-morbidities of AIDS are typically insidious, since the second infection usually develops many weeks later [19]. However, non-HIV-infected patients are more likely to have worse results of PCP treatment than HIV-positive patients due to delayed or missed diagnosis [20]. Many cases presented in the literature indicate that PCP is still a highly troublesome disease. For example, Aviles et al. presented a 3-month-old girl with subglottic hemangiomas who was admitted to the hospital with shortness of breath and progressive respiratory distress after 5 weeks of aggressive corticosteroid treatment. The third-generation cephalosporin antibiotic that was administered was not sufficient and intravenous therapy was ineffective. On Day 5 of hospitalization the patient underwent bronchoalveolar lavage. The microbiology result revealed *Pneumocystis carinii* cysts [1].

**Fig. 1:**
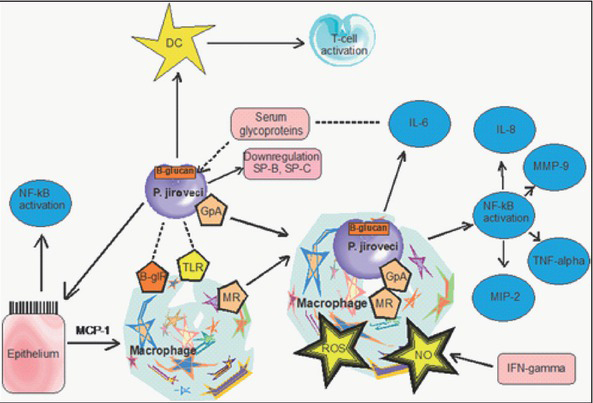
Principal interactions between *Pneumocystis jirovecii* and the respiratory tract epithelium and immune cells. DC, Dendritic cells; B-glR, β-glucan receptors; GpA, Major surface glycoprotein; IFN-gamma, interferon gamma; IL-6, interleukin 6; IL-8, interleukin 8; MCP-1, Monocyte chemoattractant protein 1; MIP -2, macrophage inflammatory protein 2; MMP-9, Matrix metalloproteinase 9; MR , Mannose receptor; NO, Nitric oxide; NF-*κ*B, Nuclear factor kappa-light-chain-enhancer of activated B cells; ROS, Reactive oxygen species; SP-B and SP-C, Surfactant proteins B and C; TLR, toll-like receptor; TNF-alpha, tumor necrosis factor alpha. Adapted from [21].

Figure 1 presents the current understanding of principal interactions between *Pneumocystis jirovecii* and the respiratory tract epithelium and immune cells of the host [21] (fig. 1).

## Diagnosis and treatment

The opportunistic species *Pneumocystis* cannot be grown in culture. The laboratory diagnosis of the pathogen’s presence in biological specimens relies on microscopic demonstration using such histological visualization methods as the Papanicolaou, toluidine blue O, Gomori methenamine silver, or Wright-Giemsa stains. Table I summarizes the clinical symptoms typical for the disorder. The presence of crazy-paving, ground-glass opacity, and centrilobular nodules is predominant in computed tomography images of the chest in PCP patients. A subpleural sparing can be found in some subjects [22]. Wakefield and colleagues were the first to point out that the PCR method is more sensitive but less specific in comparison to traditional methods of detection. In this sophisticated method, both oral washing and sputum can be used as specimens for evaluation [3]. In the Sing et al. study, PCR of BAL reached specificity and sensitivity of 100% in detecting PCP in HIV-infected patients [23]. (tab. I).

**Table I:** Grading of clinical symptoms in *Pneumocystis pneumonia*.

	Disease Exacerbation		
Clinical feature	Mild	Moderate	Severe
Dyspnea	On exertion	On minimal exertion/possibly at rest	At rest
Oxygen saturation	SaO_2_ > 96%	SaO_2_ of 91–96%	SaO_2_ < 91%
Radiologic findings	Minimal changes on CXR	Diffuse interstitial changes on CXR	Extensive interstitial changes on CXR
Other	---	Fever possible	Tachypnoea at rest, fever, cough

SaO_2_: oxygen saturation; CXR: chest radiograph.

Current recommendations indicate that the treatment of choice in patients older than 2 months of age is trimethoprim-sulfamethoxazole medication. Children should first be treated intravenously for 21 days and then orally twice p.d. as continuation. Common adverse effects during the treatment include rash, diarrhea, and hematological and hepatic abnormalities [24]. One study reported that elevated levels of lactate dehydrogenase could often be detected in peripheral blood of PCP patients and these levels were higher than those observed in patients suffering from bacterial pneumonia [25].

The second line of therapy is intravenous pentamidine. It should be used in children with clinical treatment failure after the administration of trimethoprim-sulfamethoxazole. Considerable toxicities, such as electrolyte abnormalities, hypoglycemia, pancreatitis, and even renal or cardiovascular dysfunction were observed in a fraction of children undergoing pentamidine therapy. Alternative means for cure of PCP are the following combined regimens: clindamycin plus primaquine, or trimethoprim plus dapsone, or clindamycin plus caspofungin [26]. Moreover, systemic corticosteroids are usually administered in AIDS patients with severe PCP [27].

Of clinical significance, a PCP prophylaxis with trimethoprim-sulfamethoxazole should be administered in children with AIDS-underlying illness, patients with low CD4 counts (<500 cells/μL), and infants whose mothers are HIV-infected [28]. However, the recommendation of such prophylaxis does not seem to fully resolve the problem, since studies on PCP in HIV-infected patients maintained on antiretroviral therapy and co-trimoxazole have been highlighted by a recent systematic review [29]. Other drugs used as prophylactic measures for PCP include pentamidine and atovaquone [30]. An emerging task for future research is the identification of specific mutations in *Pneumocystis jirovecii* genes responsible for the pathogen’s drug resistance, and of alternative therapies to treat PCP [4, 31].

## Conclusions

A good body of evidence, especially examining HIV-infected children, has already indicated significant problems arising from *Pneumocystis* colonization and resultant lung disorders. Future studies are needed to define the true clinical significance and epidemiology of *Pneumocystis* infections. Moreover, a further understanding of the pathogenesis of *Pneumocystis* pneumonia is of ultimate importance for successful treatment of this still life-threatening disease.
